# P-984. Assessment of Clinical Practice Gaps Negatively Impacting Vaccination Rates for Hepatitis B

**DOI:** 10.1093/ofid/ofae631.1174

**Published:** 2025-01-29

**Authors:** Sara Thorpe, Amy Larkin

**Affiliations:** Medscape Education, New York, New York; Medscape Education, New York, New York

## Abstract

**Background:**

The World Health Organization (WHO) and the US Department of Health and Human Services (HHS) have set goals for the elimination of viral hepatitis infection as a public health threat by 2030[HHS 2021; WHO 2016]. Barriers to achieving viral hepatitis elimination exist and progress toward hepatitis B (HepB) elimination has stalled. Understanding clinician gaps related to knowledge is critical to improve education and training to improve vaccination rates for HepB.

**Methods:**

A survey instrument of 25 multiple choice, knowledge- and case-based questions allowed participants to assess their clinical knowledge related to HepB vaccination. Respondent confidentiality was maintained and responses were de-identified and aggregated prior to analyses. Data collection occurred from June 23, 2023 to August 31, 2023.

**Results:**

**Table:** 

Topic	Gaps Measured with Incorrect Responses to Knowledge and Clinical Decision-Making Questions (%)		
	PCPs (N=158)	Pharmacists (N=98)	Public Health Specialists (N=36)
Knowledge			
Prevalence of chronic HepB in the US	56%	45%	53%
Prevalence of US population vaccinated against HepB	51%	38%	47%
Healthy People 2020 goal related to HepB	61%	72%	50%
HepB vaccine safety	62%	35%	39%
Continuity of care for HepB vaccines series	46%	39%	36%
Completion rates for HepB vaccine series	57%	54%	56%
Differences in completion rates for different HepB vaccines	74%	78%	64%
Seroprotection rates with HepB vaccines	56%	54%	50%
Clinical Decision-Making			
HepB vaccination recommendation in a patient case	42%	50%	31%
HepB vaccine selection in a patient case	66%	57%	56%
Contraindication for HepB vaccine	54%	59%	50%
Most common rationale for not discussing HepB vaccination	82%	84%	94%
Assessment of practice HepB vaccine coverage	59%	48%	44%
Confidence			
Self-reported confidence in ability to discuss HepB vaccines with your patients	75% not confident	81% not confident	64% not confident

**Conclusion:**

This assessment yielded important insights into gaps related to HepB vaccination rates. PCPs have lower knowledge related to HepB vaccines in many areas which may be contributing to lower HepB vaccination rates. Pharmacists are the least confident in recommending HepB vaccines to their patients, followed closely by PCPs. Additional education is needed to increase knowledge and confidence to meet elimination goals.

**Disclosures:**

**All Authors**: No reported disclosures

